# Advancing pre-exposure prophylaxis (PrEP) implementation for people who use drugs: an introduction to and lessons from the special series

**DOI:** 10.1186/s13722-025-00617-3

**Published:** 2025-10-24

**Authors:** Angela R. Bazzi, Hansel E. Tookes, Tyler S. Bartholomew

**Affiliations:** 1 Herbert Wertheim School of Public Health, University of California San Diego, 9500 Gilman Drive, La Jolla, CA 92093 USA; 2 Boston University School of Public Health, Boston, MA USA; 3 Department of Medicine, University of Miami Miller School of Medicine, Miami, FL USA; 4 Department of Public Health Sciences, University of Miami Miller School of Medicine, Miami, FL USA

## Introduction to the special series

Pre-exposure prophylaxis (PrEP) is a safe, highly effective biomedical HIV prevention tool that is tremendously underutilized among people who use drugs (PWUD), including alcohol, a group disproportionately impacted by HIV. In addition to sexual and injection-related exposure to HIV, structural stigma, criminalization, fragmented healthcare systems, and disjointed public health infrastructure limit access to evidence-based HIV prevention services for PWUD. Unfortunately, PrEP is rarely offered in settings where PWUD present for care, and PrEP initiation, retention, and persistence are major challenges. This special series in *Addiction Science & Clinical Practice* brings together 14 manuscripts—10 original research articles, three protocol papers, and one scoping review—that deepen our understanding of how to improve PrEP implementation, from delivery and uptake, through retention in varied service settings and populations of PWUD. As the series editors, below we synthesize themes that cut across the special series and conclude with recommendations for implementation, clinical practice, research, and policy.

## Persistent, multilevel barriers to PrEP access

Across this special series, papers provided rich qualitative and quantitative evidence with some emerging themes that add to existing literature on persistent, multilevel barriers to PrEP access and uptake across populations of PWUD. First, at the individual level, several studies confirmed low PrEP awareness and perceived need as challenges to PrEP uptake for PWUD. For example, Eger et al.’s analysis of data collected from people who inject drugs (PWID) in San Diego, CA, found that an astounding 75% of participants had never heard of PrEP over a decade after it was approved for use in the United States. Nevertheless, preferences were strongest for long-acting formulations, confirming that regardless of awareness, daily oral PrEP could pose challenges to initial and sustained engagement [1]. Similarly, Fletcher et al. surveyed U.S. veterans with unhealthy alcohol use and found low PrEP awareness and willingness, including among those experiencing high risk of alcohol-related harms [2]. In Uganda, Kamusiime et al. interviewed PWID accessing harm reduction services and found that, despite awareness of behaviors that elevated their HIV risk (e.g., sharing syringes, transactional sex), many study participants prioritized daily survival needs over initiating PrEP [3]. Perez-Brumer et al. provided additional insights from qualitative interviews with participants in the HIV Prevention Trials Network (HPTN) 094 INTEGRA Study, identifying low perceived risk and competing survival priorities (e.g., food, drugs, shelter) that took precedence over HIV prevention [4]. Similar barriers were identified in Harris et al.’s qualitative study with women who inject drugs presenting at a bridge clinic in Boston, MA [5].

Interpersonal and provider-level barriers also emerged in articles published in this series. In a retrospective record review, Bradford et al. found that among 300 hospitalized PWID without HIV, only two patients had documentation of PrEP-related discussions with providers, and only one patient was discharged with PrEP, suggesting that providers may have low PrEP awareness, competing priorities, or uncertainty about how to assess candidacy and prescribe PrEP in complex clinical contexts [6]. Marotta et al.’s survey of rural primary care providers found that few felt prepared to prescribe PrEP to PWUD due to lack of training and institutional support [7]. Harris et al. also interviewed bridge clinic providers, finding that some expressed discomfort and uncertainty discussing sex work-related risks with women who inject drugs. Providers also identified competing clinical priorities and a lack of PrEP adherence supports for this population as additional barriers, which reduced providers’ confidence [5].

Encouragingly, many providers recognize the importance of PrEP, and some have experience successfully prescribing to PWUD. Unsurprisingly, Pickering et al.’s survey of harm reduction and community-based organization providers found positive views of PrEP (in general) and contingency management for adherence support, especially when training is provided to help support implementation readiness of staff and organizations [8]. Rozansky et al. found that addiction consult service providers were responsible for the majority of PrEP and post-exposure prophylaxis (PEP) prescribing to hospitalized PWID [9], underscoring the value of role clarity and tasking specialized consult services with HIV prevention for PWUD when available.

Beyond the individual, interpersonal, and provider levels, most papers in the series highlighted structural factors—including housing instability and evictions, criminalization, police harassment, and systemic or structural racism [3–5, 10]—that remain major impediments to PrEP access. A persistent systems-level challenge is the limited integration of HIV services into addiction and primary care, with Bradford et al.’s study suggesting that hospitals lacked protocols to offer PrEP to PWUD even during hospitalizations from injection-related infections, which represent critical reachable moments [6]. Additionally, Marotta et al. concluded that rural clinics lacked referral networks and internal systems to support PrEP delivery [7]. Taken together, studies in this series suggest that scaling PrEP for PWUD will require structural reforms, including those that promote stable housing, legal protections, and integrated, low-threshold models of care.

## Facilitators and promising models of PrEP integration

While numerous barriers impede PrEP access among PWUD, this special series also illuminates implementation facilitators and emerging models of care focused on improving PrEP interest, uptake, retention, and adherence. In addition to bolstering patient willingness [2–4], these include empowerment and peer navigation strategies and co-locating and adapting services to leverage trusted and frequently accessed settings. First, in Goddard-Eckrich et al.’s secondary analysis of data from a randomized controlled trial engaging Black women under community supervision, participants randomized to the “Empowering African-American Women on the Road To Health” (E-WORTH) intervention had increased PrEP awareness and willingness over 12 months. These findings demonstrate the utility of this theory-based, culturally tailored intervention with community-specific messaging in empowering women in community supervision settings to consider new HIV prevention approaches [10]. Peer support and flexible, responsive care models also emerged as additional facilitators of PrEP uptake across several manuscripts. For example, Reback et al. described the protocol for the “Assistance Services Knowledge-PrEP” (A.S.K.-PrEP) intervention trial combining PrEP navigation with supportive text messaging to engage transgender women and sexual minority men with substance use disorders in the PrEP care continuum [11].

Next, one of the most effective facilitators for PrEP engagement is delivering services in settings where PWUD already feel welcome and supported. Although Merle et al.’s scoping review found that very few published interventions target structural or systems-level change, financing, or policies [12], several papers in this collection highlighted innovative ways to address these challenges. Bartholomew et al. described the protocol for Project CHARIOT, which uses a peer-facilitated telehealth approach to deliver integrated PrEP and medications for opioid use disorder (MOUD) through a syringe services program (SSP). Participants are connected to clinicians via tablets and offered PrEP and addiction services in a non-judgmental and accessible environment, minimizing barriers like stigma and transportation challenges, leveraging the fundamental principle of harm reduction by meeting PWUD where they are [13].

The mobile co-delivery of PrEP along with other critical services (e.g., wound care, addiction treatment) was also perceived to be highly acceptable to participants in the INTEGRA Study and Ugandan harm reduction service setting [3, 4]. Rozansky et al. demonstrated the value of an inpatient addiction consult service in increasing PrEP and PEP prescribing, illustrating the value of structural integration [9]. For women involved in the criminal legal system, Meyer et al.’s protocol describes a trial of the “Athena strategy” involving a PrEP decision aid and integrated delivery of PrEP and MOUD using an eHealth platform to help reduce system navigation burdens and circumvent geographic and bureaucratic fragmentation [14]. Together, these manuscripts show that PrEP uptake likely improves when services are tailored to cultural context, guided by peers with lived and living experience, embedded in trusted settings, and co-delivered with other supports to best meet patient needs.

## Leveraging implementation science to advance PrEP access

Finally, a forward-looking theme that emerged from this series relates to the use of implementation science methods to improve the delivery of PrEP—an evidence-based HIV prevention tool—to PWUD in real-world settings. Merle et al.’s scoping review was guided by the Consolidated Framework for Implementation Research (CFIR) to map PrEP implementation determinants (barriers and facilitators) for PWUD, finding significant gaps in attention to organizational and policy-level determinants, and noting that few studies focused on structural interventions to improve provider behavior or system-level readiness [12]. Perez-Brumer et al.’s study incorporated the Practical, Robust Implementation and Sustainability Model (PRISM) to analyze data from the INTEGRA Study, showing how individual characteristics, organizational context, and broader systems all interact to shape PrEP delivery and uptake, highlighting the need to address both supply- and demand-side implementation and sustainment determinants [4].

Studies in the series also used implementation science methods and study designs, including hybrid effectiveness-implementation approaches that simultaneously assess clinical effectiveness and implementation outcomes. Examples include Bartholomew et al.’s Project CHARIOT protocol to test telehealth-delivered PrEP through SSPs using systems redesign principles while measuring adoption, feasibility, and fidelity [13], and Meyer et al.’s Athena study protocol that embeds PrEP into MOUD care for women under community supervision [14]. Similarly, Reback et al.’s protocol employs a stepped-care model where navigation is followed by contingency management for participants who struggle with PrEP adherence, testing both efficacy and adaptability [11]. These trials exemplify how implementation science can guide iterative improvement and inform the most effective strategies for supporting PWUD in accessing PrEP. These protocols also promise to build this evidence base to ensure that PrEP can be successfully adopted, implemented, and sustained in diverse settings that serve this population.

## Recommendations and conclusions

Having synthesized key themes from the special series, including multilevel barriers, facilitators and promising care models, and implementation science approaches, we now turn to recommendations for clinical practice, research, and policy to capitalize on these insights and advance PrEP for PWUD. First, to improve PrEP delivery for PWUD in clinical practice, healthcare systems and providers must prioritize service integration (e.g., of infectious diseases and addiction care), accessibility of multiple PrEP modalities (including long-acting formulations), and stigma reduction. PrEP should be better integrated into settings where PWUD access care, including inpatient addiction consult services and bridge clinics, outpatient addiction treatment services, and most importantly, non-judgmental and trusted environments like SSPs and other community-based organizations. Peer navigation interventions delivered by individuals with lived and living experience and culturally tailored outreach should be core components of service delivery, especially for the most socially and structurally marginalized populations of PWUD such as Black women, transgender individuals, people involved in the criminal legal system, and those residing in resource-poor settings. Clinics should adopt low-threshold, flexible models including mobile units, telehealth, and simplified laboratory protocols. Implementation strategies should target building provider confidence in assessing HIV risk and prescribing PrEP through trauma-informed, respectful clinical interactions. Implementation of clinical decision aids, standing orders, and team-based approaches can further support routine PrEP engagement.

Future research should focus on implementation strategies and methods (e.g., hybrid effectiveness-implementation approaches) in real-world settings such as jails, rural primary care clinics, shelters, and SSPs. There is a critical need to center marginalized groups of PWUD—especially women, youth, and racial and ethnic minorities—and to involve them in participatory research processes. Implementation science frameworks should help guide study design, data collection, and analysis. Researchers should evaluate specific strategies such as contingency management, peer navigation, task-shifting, and digital adherence supports to determine their feasibility, acceptability, and scalability of PrEP for PWUD. Long-term outcomes including PrEP adherence and retention in care should be tracked alongside implementation outcomes like fidelity, reach, and cost-effectiveness.

Policy change is paramount to dismantling structural barriers and creating enabling contexts for PrEP delivery to PWUD. These policies include decriminalizing drug use and expanding support for harm reduction services, such as legal authorization and sustained funding for SSPs. Public health systems must ensure that multiple PrEP modalities—including long-acting formulations—are covered by Medicaid and accessible to all, regardless of housing status or income. Integrated payment models should incentivize bundled care that combines HIV prevention, substance use disorder treatment, harm reduction services, primary care, and behavioral health. Housing, transportation, and legal support should also be considered essential components of HIV prevention for PWUD. Investment is urgently needed in workforce development, including funding for peer navigation and provider training, and in data systems that disaggregate PrEP uptake by service delivery setting and population to assess reach. Finally, public-facing campaigns should work to reduce stigma and normalize PrEP as part of comprehensive HIV prevention for PWUD.

In conclusion, this special series on PrEP for PWUD underscores the urgent need for multi-level interventions to expand PrEP access among PWUD, a high-priority community. The series illuminates daunting challenges reinforced by persistent multilevel barriers (including stigma and limited integration of PrEP into key service settings accessed by PWUD). It also highlights tangible opportunities for evaluating and scaling up promising strategies to improve PrEP access and delivery. While newer, long-acting formulations hold promise for addressing PrEP adherence challenges, they do not inherently resolve systemic issues ranging from low patient knowledge to provider inattention, insurance limitations, and fragmented health systems. Importantly, the four protocol papers are harbingers of this transformative time in PrEP research for PWUD. In pursuit of PrEP equity, addressing implementation challenges will pave the way to improved access to PrEP for PWUD and PrEP for all.
